# Advancing clinical and translational research in germ cell tumours (GCT): recommendations from the Malignant Germ Cell International Consortium

**DOI:** 10.1038/s41416-022-02000-4

**Published:** 2022-10-13

**Authors:** Adriana Fonseca, João Lobo, Florette K. Hazard, Joanna Gell, Peter K. Nicholls, Robert S. Weiss, Lindsay Klosterkemper, Samuel L. Volchenboum, James C. Nicholson, A. Lindsay Frazier, James F. Amatruda, Aditya Bagrodia, Michelle Lockley, Matthew J. Murray

**Affiliations:** 1 Children’s National Medical Center, Washington, DC USA; 2 Cancer Biology and Epigenetics Group, Research Center of IPO Porto, RISE@CI-IPOP Health Research Network, Portuguese Oncology Institute of Porto, Porto Comprehensive Cancer Center, R. Dr. António Bernardino de Almeida, 4200-072 Porto, Portugal; 3 Department of Pathology, Portuguese Oncology Institute of Porto, R. Dr. António Bernardino de Almeida, 4200-072 Porto, Portugal; 4 Department of Pathology and Molecular Immunology, ICBAS–School of Medicine and Biomedical Sciences, University of Porto, Rua Jorge Viterbo Ferreira 228, 4050-513 Porto, Portugal; 5 Department of Pathology, Lucile Packard Children’s Hospital Stanford, Palo Alto, CA USA; 6 The Center for Cancer and Blood Disorders, Connecticut Children’s Medical Center, Hartford, CT USA; 7 Department of Pediatrics, University of Connecticut Medical School, Farmington, CT USA; 8 The Jackson Laboratory for Genomic Medicine, Farmington, CT USA; 9 Faculty of Life Sciences, University of Bradford, Bradford, UK; 10 Department of Biomedical Sciences, Cornell University College of Veterinary Medicine, Ithaca, NY USA; 11 Department of Pediatric Hematology and Oncology, Dana-Farber Cancer Institute, Boston, MA USA; 12 Department of Pediatrics, University of Chicago, Chicago, IL USA; 13 Department of Paediatric Haematology and Oncology, Cambridge University Hospitals NHS Foundation Trust, Cambridge, UK; 14 Department of Paediatrics, Level 8, Cambridge University Hospitals NHS Foundation Trust, Cambridge, UK; 15 Cancer and Blood Disease Institute, Children’s Hospital Los Angeles; Department of Pediatrics, Keck School of Medicine, University of Southern California, Los Angeles, CA USA; 16 Department of Urology, University of California San Diego, San Diego, CA USA; 17 Centre for Cancer Genomics and Computational Biology, Barts Cancer Institute, Queen Mary University of London, London, UK; 18 Department of Pathology, University of Cambridge, Cambridge, UK

**Keywords:** Germ cell tumours, Gynaecological cancer, Testicular cancer

## Abstract

Germ cell tumours (GCTs) are a heterogeneous group of rare neoplasms that present in different anatomical sites and across a wide spectrum of patient ages from birth through to adulthood. Once these strata are applied, cohort numbers become modest, hindering inferences regarding management and therapeutic advances. Moreover, patients with GCTs are treated by different medical professionals including paediatric oncologists, neuro-oncologists, medical oncologists, neurosurgeons, gynaecological oncologists, surgeons, and urologists. Silos of care have thus formed, further hampering knowledge dissemination between specialists. Dedicated biobank specimen collection is therefore critical to foster continuous growth in our understanding of similarities and differences by age, gender, and site, particularly for rare cancers such as GCTs. Here, the Malignant Germ Cell International Consortium provides a framework to create a sustainable, global research infrastructure that facilitates acquisition of tissue and liquid biopsies together with matched clinical data sets that reflect the diversity of GCTs. Such an effort would create an invaluable repository of clinical and biological data which can underpin international collaborations that span professional boundaries, translate into clinical practice, and ultimately impact patient outcomes.

## Introduction

Germ cell tumours (GCTs) are a heterogeneous group of neoplasms affecting patients of all ages, including neonates, children, adolescents, and adults. GCTs have two distinct epidemiological peaks, one in early childhood [0–4 years of age (y)] and a second peak that starts in adolescence. GCTs account for only 3% of tumours in children <15 y, with an incidence of 0.5 per 100,000 [1]. In contrast, GCTs are the most common solid malignancy in adolescents and young adult (AYA) patients [2], accounting for 15% of extracranial tumours between the ages of 15–29 y, with an incidence in the US of 11.4 per 100,000 in adolescent males [3].

Historically, GCTs have been classified based on their site of origin as gonadal (including testicular and ovarian tumours) or extragonadal. Extragonadal tumours are further segregated into extracranial GCTs (including mediastinal, retroperitoneal, and sacrococcygeal sites) and intracranial GCTs (most frequently located in the suprasellar region and pineal gland) [4]. Testicular GCTs (TGCTs) are the most common solid neoplasm in young adult males aged 15–44 y [5]; in Europe, these have an overall crude incidence rate of 30.9 per million per year [6]. In contrast, ovarian GCTs are exceedingly rare, accounting for an average of 2.9% (range: 1.3–5.0%) of all ovarian tumours [7], with a rate of 0.7 per million per year in Europe [6]. Extragonadal extracranial GCTs are similarly rare, with a rate of 0.7 per million per year in Europe [6] Finally, intracranial GCTs represent just 1% of primary brain tumours in children and AYA in North America, with an overall incidence of 0.6 per million per year [8], and 0.4 in Europe [6]. Despite the higher prevalence of GCTs in AYA, when segregated by location and age-group, these tumours become relatively rare, resulting in substantial barriers to translation of knowledge.

GCTs are unique neoplasms, recapitulating phenomena that take place during embryonic germ cell development [4, 9]. Additionally, GCTs are biologically and histologically diverse, encompassing the full spectrum of both benign and malignant disease. Despite their complexity, major advances have been made in our understanding of the biology of GCTs over the last decade [reviewed in [4, 10–13]]. However, most knowledge has been derived from TGCTs of the young adult [14–16] treated in Northern/Western countries. Therefore, we lack similar biological insights into less frequently encountered histological subtypes, age-groups, and/or sites. This gap in knowledge has translated into fewer translational gains [17, 18] and lack of novel evidence-based treatment approaches [19–21] in these less represented GCT groups. Recent advances in culturing primordial germ cells and developing animal models of GCTs provide new opportunities for pre-clinical testing of novel therapeutic approaches. Further progress in creating accurate and accessible experimental GCT models will be important, in parallel with efforts focused on clinical specimens as described here, to fully accelerate translational studies aimed at improving clinical outcomes.

Although biobanks are operating in several regions, with some being dedicated to specific cancer types (e.g., breast, lung, and colorectal cancer) or or medical conditions (e.g., brain, cardiovascular, and kidney disease), dedicated GCT biobanks are not available [22, 23]. The discovery of clinically relevant GCT biomarkers and their implementation into routine clinical care has been stymied by the absence of a co-ordinated approach to tackle the challenges of low prevalence and diversity of tumour phenotypes [17, 18]. Further barriers include the inconsistent use of terminology, complex classification systems, and difficulties in central reporting for these tumours, which have impeded the creation of representative and clinically valuable biobanks and further hampered communication between the multiple professional groups that provide care for these patients [24, 25].

The Malignant Germ Cell International Consortium (MaGIC; https://magicconsortium.com/) was created to address these barriers, by bringing together a wide range of specialists from multiple cooperative groups, promoting research initiatives, and providing evidence-based approaches to the management of patients with GCTs. A major breakthrough in clinical data collection has been achieved through international collaborative efforts and led to the creation of the GCT data commons, developed within the Pediatric Cancer Data Commons by MaGIC (see ‘Data annotation’ section below), thus increasing the opportunities for research in rare diseases such as GCTs [15, 24, 26, 27]. However, matching biospecimens are frequently lacking. The creation of dedicated GCT biobanks would complement the efforts already made and would provide an invaluable resource. In our view, such comprehensive GCT biobanks can only be achieved through international cooperation between centres, allowing for acquisition of high-quality clinical data and matching biospecimens from diverse sources [28, 29].

**Fig. 1:**
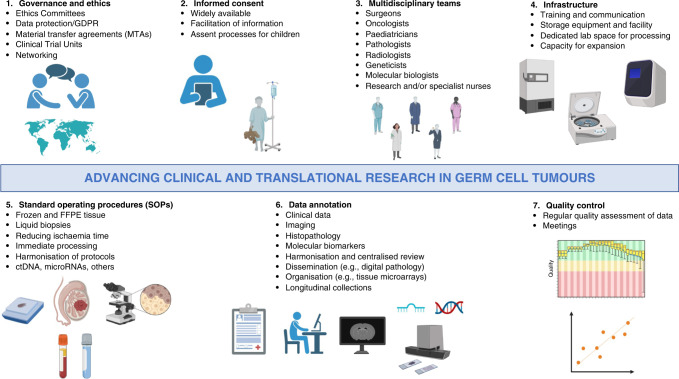
Overview of an integrated biobanking strategy for germ cell tumour (GCT) biospecimens, and related challenges. Key: ctDNA circulating tumour DNA, FFPE formalin-fixed paraffin-embedded, GDPR General Data Protection Regulation, MTA Material Transfer Agreement, SOP Standard Operating Procedure. Created with BioRender.com.

Here, we present a framework envisaging the ideal global biobank to further this mission (Fig. 1). Despite being aware that such optimal conditions may be hard to achieve and organise in practice, our aim is to propose a collaborative initiative to acquire, share, and investigate pathological, radiological, and clinico-demographic data of these rare tumours in an inclusive manner. Therefore, smaller ‘best practice’ contributions from centres with limited resources and numbers would be welcomed and instrumental to generate the intended clinically valuable data representative of all GCT phenotypes. We anticipate that such collaborative efforts will substantially improve our current knowledge and provide a platform to interrogate the most pressing questions in the management of these tumours.

From this point, we will follow the typical clinical journey for patients with GCTs, from presentation, through treatment, and into follow-up. We will highlight specific time points that represent opportunities to acquire and store invaluable data and biospecimens prospectively. If successful, this approach will offer a unique opportunity to make a major and sustainable impact in advancing the management of these patients.

## Search strategy and selection criteria

References for this Policy Review were identified through PubMed search, using the search terms ‘germ cell tumours’, ‘biobanks’, ‘research’, ‘data commons’, ‘informed consent’ and ‘standard operating procedures’, including all entries until 2022. Articles were also selected through searching the authors’ own files. Only papers published in English were included. The final reference list was generated based on originality and relevance to the broad scope of this Policy Review.

## Governance and ethics (Fig. 1(1))

The establishment of organised and dedicated biobanks for systematic collection and appropriate storage of data from these patients allows the building of research networks, fosters international cooperation and initiation of multi-institutional studies and trials, increasing substantially the chances of achieving clinically meaningful findings. However, the creation of a resource-rich infrastructure for translational research is not an easy task. It requires commitment and close collaboration between clinicians, researchers, and institutions. The development of a clear governance structure that prioritises the utilisation of biospecimens and provides accessibility to the data to all collaborators is of paramount importance [30].

Close collaboration with local ethics committees is important to approve the banking protocols and ensure the informed consent meets institutional standards [31]. The recently adopted General Data Protection Regulation (GDPR) has introduced new challenges for research in Europe, particularly relating to biobanking and data sharing [32]. Scientific directors of biobanks and ethics committees must be well-informed about all intended research projects and safeguard the compliance of all ethical requirements. Additionally, to foster international collaborations and data sharing across centres, effective strategies for data protection and material transfer agreements (MTAs) should be in place. Specifically, ethics committees and MTAs should be pre-prepared to enable a rapid response to collaboration and project grant requests. Finally, clinical trials units should also be involved, in the event a discovery prompts an early-phase clinical trial.

## Informed consent (Fig. 1(2))

One of the first steps to establish a biorepository biobank is the development of an informed consent process that can be widely available to all the institutions and specialties involved in the care of GCT patients [33]. Such informed consent should be easily accessible to all practitioners involved in clinical care of these patients. Strategies for having a specific time-point within the patient consult to discuss biobanking should be planned ahead, but can be challenging given the clinical caseload. These should be adapted to fit the healthcare practice setting of each institute. Additional challenges in GCTs involve the inclusion of patients <16 y and consent given by parents or other relatives, which must be addressed in study planning [34]. The international cooperative model we propose aims to provide experiences and support institutions with organisation of these ethical aspects, so that individual ‘best practice’ contributions can be gathered from several settings.

## Multidisciplinary teams (MDTs) (Fig. 1(3))

The creation of well annotated biobanks involves close collaboration between clinicians, researchers, institutions and patients alike [35]. Since GCTs can present in different locations, collaboration across subspecialities is imperative to avoid pitfalls in diagnosis, staging and treatment. Additionally, nuances in the management of patients with GCTs may require referral to centres of excellence and expert review to achieve superior outcomes [36]. This also highlights the advantages of dedicated multidisciplinary tumour (MDT) boards, including at the multi-institutional level [37]. This collaborative approach has been shown to improve the outcomes and refine treatments of GCT patients [25]. In our view, adopting such an effort for acquisition of GCT patient data may have the added benefit of improving communication and sharing of perspectives between different subspecialities, both within each institution and also among institutions.

## Infrastructure (Fig. 1(4))

Successful biobanks require a solid infrastructure to obtain, store, and share biospecimens. Personnel involved in biobanking must all receive training, addressing all aspects of data acquisition, from patient consent, to processing and storage [38]. Communication is important for clarification of procedures/protocols and rapid on-site sample collection and processing. For this, adequate infrastructures are needed, including a specific laboratory, storage space (with extra space always prepared in the event of possible malfunction of a freezer, for instance), centrifuges, and reagents and consumables. This may not be possible to organise in smaller institutions; however, in the biobank model we propose, organisational systems that allow for patient consent to acquire clinico-demographic data retrospectively are also welcomed, since they will add valuable information about these rare tumours. Therefore, collection of biospecimens, whilst highly desirable, would not be a requirement for participating in this effort, and could be centralised in larger institutions with wider access to laboratory research facilities.

## Standard operating procedures (SOPs) (Fig. 1(5))

### Tissue

Where feasible, the collection of tissue from surgical resections and biopsies should always be pursued. SOPs are available but rapidly changing [39]. An SOP for biospecimen collection developed by GCT experts has been developed, which is updated as required, and available via the MaGIC website (https://magicconsortium.com/magic-research/biologic-research) to facilitate the standardisation of biobanking procedures. Involving dedicated GCT pathologists with expertise in classifying these challenging tumours is also important. Engagement of the surgical and pathology team is critical to allow the appropriate preservation of tissue. The pathology team on operating theatre/frozen section duty should be alerted upfront regarding date and timing of specimens that require tissue collection for the biobank, since specimen handling is different for cases requiring collection and freezing (versus specimens intended solely for diagnostics). Specimens should be delivered immediately to pathology to reduce ischaemia prior to processing/freezing, allowing for storage of high-quality biological material. Collection of tumour tissue (without obvious necrosis) and adjacent normal organ tissue should be performed (guaranteeing that diagnosis and staging of the patient is not compromised). One strategy is to bisect the tissue fragment on its longest axis, keeping one half for snap freezing and the other ‘twin’ fragment for formalin fixation and paraffin embedding (FFPE) [39]. Tissues should be placed in appropriate containers (cassettes, cryomolds, cryotubes), appropriately labelled (with local biobank coding system and preferably with barcoding for sample tracking) and snap frozen (with various system options, including commercially available dedicated equipment). Importantly, histological confirmation of tumour presence and demarcation of the tumour area on haematoxylin and eosin (H&E) slides is fundamental for downstream nucleic acid extraction. Tumour cell content, stromal proportion and necrosis extent should be assessed on histological slides before pursuing expensive downstream molecular studies [40]. Furthermore, for FFPE tissue, annotation of time of entering and exiting 10% phosphate-buffered formalin (15–20 times more volume compared with tissue volume) at room temperature is crucial for biomarker studies (6–8 h minimum and 72 h maximum) to minimise false-positive and false-negative immunohistochemistry results.

### Liquid biopsies

Systematic biobanking of bodily fluids [blood—plasma and/or serum—but also urine, cerebrospinal fluid (CSF), semen, saliva, effusions] is key to facilitate biomarker studies. Universal, harmonised SOPs for professionals and centres are required to allow comparison and/or collation of downstream studies [41, 42]. Different pipelines (e.g., using different collection tubes or centrifugation speed) are used depending on the downstream analyses. Blood samples should be processed rapidly [typically, maximum 4 h from collection for gel separator tubes or 1 h for ethylenediamine tetraacetic acid (EDTA) tubes] [43]. For circulating tumour DNA (ctDNA) analyses, plasma is the desired source as it has a higher ctDNA yield than serum, and an additional high-speed centrifuging step should be performed to remove genomic DNA contamination. Freeze–thaw cycles should be minimised, and aliquoting in 1 ml volumes is recommended, for preserving maximum ctDNA quality [44]. For microRNA studies, serum is typically preferred; if only plasma is retrieved, a low-speed centrifugation is recommended for extracting microRNAs, while the remainder of the sample is kept aside for undergoing a second high-speed centrifugation for optimising ctDNA extraction [45, 46]. Any obvious visibly haemolysed samples should be discarded, and replacement samples sought if feasible, as substantial haemolysis may interfere with biomarker detection [47].

## Data annotation (Fig. 1(6))

The collection of accurate and high-quality clinical data is imperative for the continuous development of translational strategies in GCTs. One of the biggest challenges in clinical data acquisition is the lack of standardised collection preventing appropriate analysis of data and interpretation of results [48]. To address those barriers, the Pediatric Cancer Data Commons (PCDC, https://commons.cri.uchicago.edu/magic/) has created a platform to easily collect and harmonise data from various sources, thus providing larger samples sizes and data sets with improved power. The GCT-PCDC was designed in collaboration with GCT experts from MaGIC to overcome the sparsity of, and lack of interoperability between, data sets. Standardised data dictionaries include demographics, disease characteristics, pathology (capturing both institutional and central reviews, if available), serum tumour markers (STMs), circulating microRNA levels, surgery, chemotherapy, radiotherapy, radiological response, relapse, second malignant neoplasms, and death or follow-up, using a standardised and controlled terminology that facilitates the crosstalk between the GCT-PCDC data model and other data models/standards. Within this framework, individual institutes across the globe can apply to contribute. The GCT-PCDC will be publically available and open to all, fostering retrospective clinically-oriented research projects, such as service evaluations, audits, and associations of clinical outcome with different clinico-pathological correlates. This approach will maximise learning opportunities as widely as possible across the field. While fostering such inclusive collaboration, it should be borne in mind that for prospective treatment of patients with rare cancers, such as GCTs, centralisation of care, where feasible, in large reference centres with expertise and experience of managing high volumes of patients, is associated with improved outcomes [6].

### Diagnosis—tissue

Using the GCT-PCDC model, we encourage the future longitudinal collection of data and matching biospecimens with the overarching goal of increasing the possibility to create models to further study tumours [e.g., cell lines, organoids, patient-derived xenografts (PDXs)]. Systematic annotation of histopathological parameters of GCTs is also needed, including information about histological subtypes and staging following the most recent recommendations of the World Health Organisation (WHO) and American Joint Committee on Cancer (AJCC) [49]. When pursuing multi-institutional projects, centralised pathology review is beneficial. For example, reporting discrepancies between original and central expert opinions regarding GCT histological subtype, lymphovascular invasion, and pathological stage occurred in 31, 22, and 23% of cases, respectively [49, 50]. Digital pathology is likely to become an important method of facilitating data sharing among centres for this purpose, besides being useful for its algorithms of analysis which open further research opportunities [51, 52]. Building of representative tissue-microarrays could also be instrumental to validate novel protein biomarkers by immunohistochemistry in a simple and practical manner.

### Diagnosis—body fluids

The determination of the STMs alpha-fetoprotein (AFP), human chorionic gonadotropin (HCG) and lactate dehydrogenase (LDH) are important for GCT diagnosis and prognostic purposes, as well as a follow-up tool to evaluate response to therapy [53, 54]. For TGCTs, they are also part of the risk classification system [49], and in GCTs in general as part of the International Germ Cell Cancer Collaborative Group (IGCCCG) classification [55]. Since these classical STMs are routinely collected for follow-up of GCT patients [56], drawing additional bodily fluid at those timings for biobanking will have two main advantages: avoiding extra collections for biobanking only; allowing for parallel comparison of investigational biomarkers with the standard-of-care routine biomarkers. Nevertheless, the longitudinal collection of STM levels in blood and CSF has not typically been performed until very recent prospective clinical trials [57]. Body fluid storage for biomarker studies is an area requiring close communication and collaboration. However, given the limitations of classical STMs, reviewed elsewhere [53], it is worth investing in as it could potentially transform management for GCT patients. Of note, microRNAs, in particular miR-371a-3p [58, 59], have emerged as promising non-invasive biomarkers for GCT patients, outperforming STMs for diagnosis, risk-stratification and follow-up [60–62]. These are therefore likely to become established in clinical practice in due course, alongside existing STMs [41].

Ideally, matched collections of relevant clinical data, imaging, and tissue and liquid biopsies are collected from the same patient. Getting systematic data across time, during patients’ follow-up and hospital visits, enables the study of prognostic biomarkers, minimal residual disease (MRD) investigations and prediction of sensitivity and resistance to therapy, as well as related toxicity. Including healthy blood donor volunteers visiting blood banks as a control population, as much as possible age- and gender-matched to the study population, could be envisaged and needs further informed consent and related procedures. For GCTs, international (biobank) collaboration is the only practical way to effectively study important clinical questions such as cisplatin resistance, given the overall good outcomes for patients from this disease. Collections of metastatic and/or refractory/relapsed tumour samples and corresponding liquid biopsies from these patients, represents an important approach to study cisplatin resistance [63]. It may also allow the development of in vitro and in vivo models for studying the disease in the lab, by creating patient-derived organoid or PDX models [64]. Examples of potential avenues of investigation made possible by collaborative GCT biobanks and data commons are provided in Table 1.

**Table 1:** Examples of investigations made possible by a collaborative germ cell tumour (GCT) biobank and/or clinical trials.

Scientific investigation	Theme	Scientific question	Patient groups	Potential patient/clinical benefit
Cisplatin resistance	Basic Biology	Understand the biology and emergence of resistance	Particularly for intermediate- and high-risk GCT patients	• Improved and earlier identification of patients with cisplatin resistance • Opportunities for uncovering new targeted treatments
	Biomarkers	Identify tissue and liquid biopsy predictive biomarkers		
	Therapeutics	Identify genes and pathways associated with resistance		
Circulating microRNAs for extracranial GCTs	Biomarkers	Improve sensitivity of assay further	Particularly for patients without existing tumour markers e.g., TGCT seminoma patients	• Localised (stage I) disease: improved and earlier identification of patients with occult micrometastatic disease following orchidectomy and/or relapse • Reduced requirement for longitudinal serial CT scans in follow-up
Circulating microRNAs for intracranial GCTs	Biomarkers	Use CSF and serum microRNAs to segregate malignant GCTs from other brain tumours and identify GCT subtypes	Patients with midline brain tumours	• Identify malignant GCT diagnosis • Need to identify second microRNA panel to segregate malignant GCT subtypes for risk-stratification and treatment • Ultimate aim for non-invasive diagnosis and prognostication
Circulating tumour DNA (ctDNA) and circulating tumour cells (CTCs) for GCTs	Biomarkers	Identify novel diagnostic, prognostic, and monitoring biomarkers (e.g., DNA methylation)	Particularly for intermediate- and high-risk GCT patients, for detection of minimal residual disease (MRD)	• Improved combined biomarker panels, complementing classic serum tumour markers (STMs) and microRNAs
Teratoma	Basic Biology	Understand the biology (immature teratoma) and the emergence of somatic malignant transformation and teratoma growing syndrome (mature teratoma)	Particularly for GCT patients treated with chemotherapy (mature teratoma) and all immature teratoma patients	• Improved and earlier identification of these events associated with cisplatin resistance • Opportunities for uncovering new targeted treatments • Identify risk factors associated with recurrence of ovarian immature teratoma
	Biomarkers	Identify liquid biopsy biomarkers of teratoma		• Specific discrimination of mature teratoma from necrosis/fibrosis and viable GCT in residual masses
	Pathology	Assess the clinical significance of immature teratoma grading and/or staging in ovarian GCTs	Ovarian GCTs (immature teratoma)	• Identifying a more reproducible and robust grading system
Disease models	Basic Biology	Develop novel informative models, representative of GCTs (e.g., organoids, patient-derived xenografts)	Representative of the whole clinical spectrum of GCTs	• Opportunities for uncovering and testing new biomarkers and targeted treatments
Radiomic/Radiogenomic studies	Diagnostics	Develop novel diagnostic algorithms	Particularly for GCT patients with metastatic disease treated with chemotherapy	• Specific discrimination of mature teratoma from necrosis/fibrosis and viable/malignant GCT in residual masses
Histopathology	Biomarkers	Identify novel tissue prognostic biomarkers (e.g., immunohistochemistry, in situ hybridisation)	Particularly for stage I GCT patients	• Better risk stratification of stage I disease (active surveillance versus adjuvant treatment)
	Pathology	Improve reproducibility of histopathological variables (e.g., digital pathology and artificial intelligence)		
Epidemiology	Risk factors	Investigate disease associations and risk factors for emergence of GCTs	Whole clinical spectrum of GCTs, particularly under-investigated non-testicular sites	• Better understand prevalence of reported disease associations (e.g., marijuana consumption)
Surgery	Therapeutics	Investigate the impact of different RPLND protocols	GCT patients with metastatic masses	• Improved referral of patients for RPLND and choice of appropriate technique

*CSF* cerebrospinal fluid, *RPLND* retroperitoneal lymph node dissection.

## Quality control (Fig. 1(7))

Finally, quality control procedures and constant re-assessment of SOPs are key to achieve standardisation and assurances, with comparable data in multicentre studies [65]. Pre-analytical variables are known to influence research findings [43] and therefore how biospecimens are collected, processed and stored, and their associated clinicopathological data, should be harmonised and well annotated, to allow all such samples/cases to be considered suitable for downstream analyses/interrogation.

## Conclusion

In summary, GCTs are rare neooplasms, characterised by substantial clinical, pathological, and biological heterogeneity. MDTs of dedicated professionals are necessarily involved in the care of these patients. Clinically meaningful observations and discoveries that improve the management of GCT patients are potentiated if multiple individuals and institutions come together and share expertise. Dedicated biobanks are critical to support the translational studies that will underpin such learning. However, many challenges lie ahead, related to universalised cataloguing and storing of GCT biospecimens (tissues and bodily fluid) and associated clinicopathological data, with appropriate governance. We present the MaGIC view of such a potential integrated infrastructure, with the aim to facilitate truly innovative and meaningful GCT research across the full spectrum of disease encountered in clinical practice, which will lead to improved outcomes for all patients affected by this orphan disease.

## Supplementary information

Checklist
